# Fucoidan as a Marine Anticancer Agent in Preclinical Development

**DOI:** 10.3390/md12020851

**Published:** 2014-01-28

**Authors:** Jong-Young Kwak

**Affiliations:** Department of Biochemistry, School of Medicine and Immune-Network Pioneer Research Center, Dong-A University, 32, Daesingongwon-ro, Seo-gu, Busan 602-714, Korea; E-Mail: jykwak@dau.ac.kr; Tel.: +82-51-240-2856; Fax: +82-51-241-6940

**Keywords:** fucoidan, anticancer, preclinical, anticancer immunity, antiangiogenesis

## Abstract

Fucoidan is a fucose-containing sulfated polysaccharide derived from brown seaweeds, crude extracts of which are commercially available as nutritional supplements. Recent studies have demonstrated antiproliferative, antiangiogenic, and anticancer properties of fucoidan *in vitro*. Accordingly, the anticancer effects of fucoidan have been shown to vary depending on its structure, while it can target multiple receptors or signaling molecules in various cell types, including tumor cells and immune cells. Low toxicity and the *in vitro* effects of fucoidan mentioned above make it a suitable agent for cancer prevention or treatment. However, preclinical development of natural marine products requires *in vivo* examination of purified compounds in animal tumor models. This review discusses the effects of systemic and local administration of fucoidan on tumor growth, angiogenesis, and immune reaction and whether *in vivo* and *in vitro* results are likely applicable to the development of fucoidan as a marine anticancer drug.

## 1. Introduction

Fucoidan is a polysaccharide that consists of sulfated fucose residues. The richest sources of fucoidan are marine organisms, including brown algae species such as *Laminaria* and *Fucus* [1]. Fucoidan-rich brown algae have been marketed as a dietary supplement or nutraceutical. Fucoidan has advantages of low toxicity, oral bioavailability, and multiple mechanisms of action. Pharmacologically, fucoidan affects many pathophysiological processes, including inflammation, vascular physiology, carcinogenesis, and oxidative stress [2,3]. Furthermore, fucoidan can easily be extracted using either hot water or acidic solutions [4]. Thus, fucoidan-containing food supplements or drinks have been traditionally administered to cancer patients in Korea, Japan, China, and other countries. Although the underlying anticancer effects of fucoidan are largely unknown, it can directly induce cytotoxicity and apoptosis in cancer cells [5]. Fucoidan can also affect cancer cells indirectly e.g., as an antiangiogenic agent. Furthermore, fucoidan has immune-stimulating effects on dendritic cells (DCs) [6,7,8,9] and natural killer (NK) cells [10,11]. Thus, fucoidan can enhance anticancer immunity through immune cell activation and influx and stimulation of the production of anticancer cytokines.

Fucoidan has been reported to be effective *in vivo* upon oral, intraperitoneal, or intravenous administration (Table 1). Australian groups have reported clinical trials using fucoidan [12,13,14,15]. Recently, Fitton reviewed the potential therapeutic use of fucoidan in various diseases, including infection, chronic inflammation and fibrosis, liver diseases, arthritis, and radiation injury [2]. Biological activities of fucoidan, including anticancer activity, may vary depending on the source of seaweed, compositional and structural traits, charge density, distribution, bonding of the sulfate substitutions, and purity of the fucoidan preparation [4,16]. 

The structure and function relationships of fucoidan and other sulfated polysaccharides have been reviewed previously [1,3,16]. This review discusses the preclinical development and use of fucoidan as a marine anticancer agent, based on *in vivo* findings in animal models of cancers or other diseases.

## 2. Cancer Cell Apoptosis *in Vitro*

Although the mechanisms underlying the antitumor activity of fucoidan are diverse, it has antitumor activity by inducing apoptosis in cancer cells. Fucoidan-mediated apoptosis of cancer cells likely involves up-regulation or down-regulation of multiple signaling pathways. However, signaling pathways leading to the apoptosis of cancer cells by fucoidan have not been fully characterized [5]. Recently, *in vitro* studies have shown the molecular mechanisms of fucoidan in the induction of apoptosis in various human cancer cells, including HL-60, NB4, THP-1, and U937 leukemic cells [17,18], MCF-7 breast cancer cells [19], AGS human gastric adenocarcinoma cells [20], A549 lung carcinoma cells [21], PC-3 prostatic cancer cells [22], and SMMC-7721 hepatocellular carcinoma cells [23]. Xue *et al.* demonstrated that intraperitoneally injected crude extracts of *Fucus vesiculosus* induced apoptosis of 4T1 breast cancer cells in tumor-bearing mice, but fucoidan alone did not cause apoptosis of some other cancer cells *in vitro* [24]. Thinh *et al.* demonstrated that highly purified fucoidan derived from the brown algae *Sargassum mcclurei* was less cytotoxic, but inhibited colony formation in DLD-1 colon cancer cells when used at up to 200 µg/mL for 48 h [25]. We showed previously that fucoidan derived from *Fucus vesiculosus* failed to induce apoptosis in K562 erythroleukemic [17] and mouse CT26 colon cancer cells [26], while it inhibited cell proliferation. These results suggest that apoptotic activity of fucoidan on cancer cells may be cell type specific.

**Table 1:** *In vivo* effect of fucoidan on tumor growth in tumor-bearing mice.

Fucoidan	Route/Dose	Tumor Type	Action Mechanism	References
*Cladosiphon okamuranus*	p.o. 5 g/kg	26 colon cancer cells	Natural killer (NK) cell-mediated	[11]
*Fucus vesiculosus* (Sigma, St. Louis, MO, USA)	i.p. 5 mg/kg	4T1 breast cancer cells	Inhibition of angiogenesis and induction of apoptosis	[24,25]
*Fucus evanescence*	10 mg/kg	Lewis lung carcinoma cells	Unknown	[27]
From Ze Lang Nanjing Med. Tech Co.	i.p. 200 mg/kg	Bel-7402 hepatocellular carcinoma in nude mice	Inhibition of proliferation	[28]
*Fucus vesiculosus* (Sigma, St. Louis, MO, USA)	Foot-pad injection 0.25 mg/mice	4T1-xenograft mice	Prevention of metastasis	[29]
*Sargassum plagiophyllum*	p.o. 75 mg/kg	Diethylnitrosamine-induced hepatocellular carcinoma	Inhibition of carcinogen metabolism	[30]
*Cladosiphon okamuranus* Tokida	p.o. 100 mg/kg	Sarcoma 180 (S-180)-xenograft	Delayed tumor growth by nitric oxide produced by macrophages	[31]
*Undaria pinnatifida*	Diet containing 1% Mekabu (34 mg/day)	A20 leukemia cells	Cytolytic activity by NK cell activation	[32]
*Fucus vesiculosus* (Sigma, St. Louis, MO, USA)	i.v. 5 mg/kg	Lewis lung carcinoma cells B16 melanoma cells	Antiangiogenic effect	[33]
*Ascophyllum nodosum*	1 mg/mice	MOPC-315 plasma cell tumor	Prevention of angiogenesis in tumor tissues	[34]

## 3. *In Vivo* Anticancer Effects

Tumor-bearing animal models are commonly utilized to study the effects of therapeutic interventions. Recently, fucoidan was shown to inhibit the growth of tumor cells in several animal models (Table 1). Importantly, fucoidan treatment is relatively safe in animals. For example, tumor-bearing mice tolerated repeated injections of a moderate dose of fucoidan (10 mg/kg) [27]. However, fucoidan at 25 mg/kg caused toxicity in the same mouse model [27]. When fucoidan derived from *Laminaria japonica* was administered to rats orally at 300 mg/kg per day, there were no significant toxic effects [35]. Other groups also demonstrated that there was no difference in body weight between controls and animals treated with 200 mg/kg of fucoidan administered intraperitoneally [28]. Importantly, in a recent phase-two clinical trial in humans conducted by Myers *et al.*, a seaweed-derived nutrient supplement, containing 75 mg fucoidan plus vitamin B6, zinc, and manganese, was found to be safe when taken orally over four weeks [15]. These results suggest that fucoidan generally has low toxicity and is well tolerated.

A substantial number of animal studies have been conducted for the treatment of cancer using fucoidan. Alekseyenko *et al.* showed that while a single injection of fucoidan at 25 mg/kg failed to inhibit tumor growth in mice with transplanted Lewis lung adenocarcinoma, repeated injections of fucoidan at 10 mg/kg resulted in pronounced antitumor and antimetastatic effects [27]. In a hepatocellular carcinoma xenograft mouse model established by implanting Bel-7402 cells in nu/nu mice, administering fucoidan at 200 mg/kg intraperitoneally caused an anticancer effect partly by inhibiting the proliferation of cancer cells *in vivo*, but not by apoptosis [28]. Xue *et al.* investigated the effects of fucoidan on the metastasis of cancer cells. They demonstrated that crude extracts of fucoidan suppressed lung metastasis of 4T1 breast cancer cells as well as tumor growth [24,36]. Fucoidan markedly reduced the growth rate of 4T1 cells and significantly diminished the number of metastatic tumor nodules present in the lungs of 4T1-xenografted mice [29]. Collectively, these results suggest that fucoidan treatment suppresses tumorigenesis and metastasis, supporting the potential development of fucoidan as an anticancer drug.

Molecular mechanisms underlying the mode of action of fucoidan were studied *in vivo*. Fucoidan was found to prevent diethylnitrosamine-induced hepatocarcinogenesis by inhibiting metabolic activation of the carcinogen [30]. Takeda *et al.* found that oral administration of fucoidan effectively inhibited growth of implanted Sarcoma-180 cells in xenograft mouse models [31]. Fucoidan likely mediated nitric oxide (NO) release by stimulated macrophages in the tumor microenvironment, thus causing apoptosis. Furthermore, supernatants from fucoidan-stimulated macrophages were found to cause apoptosis of Sarcoma-180 cells. This effect was negated by the addition of a NO synthase inhibitor, N^G^-nitro-l-arginine methyl ester (l-NAME) [31]. Xue *et al.* demonstrated that β-catenin expression in tumor lesions was decreased significantly by fucoidan treatment of tumor-bearing mice [36]. In addition, fucoidan treatment lowered expression of cyclin D1 and c-myc *in vivo*. Similar to the *in vivo* results, *in vitro* experimental results also demonstrated that fucoidan modulated the Wnt/β-catenin signaling pathway. These results suggest that fucoidan exerts anticancer activity at least partly by down-regulating β-catenin signaling both *in vitro* and *in vivo*. 

It has been suggested that differing *in vivo* effects of fucoidans may depend on electrical charge density, extent of sulfonation, and the molecular weight of different preparations [37]. The requirement for continuous administration may limit the use of high-molecular-weight (HMW) fucoidan in pharmaceutical and clinical applications [38]. The *in vitro* anticancer activity of fucoidans derived from the sporophyll of *Undaria pinnatifida* was significantly higher for low-molecular-weight fucoidan (490 kDa) than for native fucoidan of 5100 kDa [39]. Azuma *et al.* further investigated molecular-weight dependent effects of fucoidan on tumor growth and survival time in tumor-bearing mouse models [11]. They showed that oral administration of fucoidan extracted from *Cladosiphon okamuranus* in tumor-bearing mice suppressed colon tumor growth, but the tumor weight was lowered in mice treated with either LMW (6.4–40 kDa) or HMW fucoidan (300–330 kDa) preparations. Therefore, studying the anticancer efficacy of LMW and HMW fucoidan preparations in animal models should be an area of future research efforts.

## 4. *In Vivo* Anticancer Immune Responses

Maruyama *et al.* studied mice that were fed a diet containing Mekabu fucoidan, derived from the sporophyll of *Undaria pinnatifida* [32]. Mice were on the diet for 10 days before subcutaneous inoculation with lymphoma cells. Thereafter, the mice were fed the same diet for 40 days. Cell growth was significantly inhibited in these mice. However, tumor growth was not inhibited in mice fed with fucoidan diet only 40 days after inoculation of lymphoma cells [32]. The authors suggested that this anticancer activity may have been due to activation of an immune response initiated even before inoculation of cancerous cells rather than a direct cytotoxic effect by fucoidan on lymphoma cells. Further *in vivo* studies have elucidated potential mechanisms underlying the fucoidan-mediated anticancer immune responses. For example, fucoidan can affect immune cell activity and cytokine production. The killer activities of T cell-mediated NK cells were enhanced in mice fed with fucoidan compared to control mice [10,32]. NK cell activation was associated with increased production of interferon (IFN)-γ and interleukin (IL)-12 by splenic T cells in fucoidan-fed mice [32,40]. Furthermore, oral administration of fucoidan increased the number of splenic NK cells in tumor-bearing mice [11]. 

It has been suggested that immunostimulation and immunosuppression occur simultaneously in cancer patients, and deactivation of certain cytokines could offer an unexploited and novel anticancer treatment approach [41]. Cancer-induced immune suppression is related to a defective IL-12–IFN-γHLA-DR axis [41]. Proinflammatory cytokines such as IL-12 are essential for inducing the Th1 response [42]. *In vitro* experiments in our laboratory have shown that the secretion of IL-12p70 and IFN-γ is enhanced by co-culturing T cells with fucoidan-activated human peripheral blood DCs, whereas fucoidan-activated DCs alone failed to produce IL-12p70 [8]. On the other hand, Hu *et al.* demonstrated that fucoidan could enhance the maturation of DCs and the cross-presentation of cancer testis antigen, NY-ESO-1 to CD8^+^ T cells, thus augmenting the cytotoxicity of T cells against NY-ESO-1-expressing cancer cells [9]. Interestingly, fucoidan was found to increase NY-SEO-1 binding to human DCs [9]. Cancer immunotherapy using DCs generated *in vitro* has been proven to be safe in clinical trials in combination with a DC activator [43]. Accordingly, we observed that the co-administration of DCs and fucoidan in tumor-bearing mice significantly decreased tumor growth when compared to the administration of DCs or fucoidan alone (in preparation). Therefore, fucoidan could modulate anticancer immune responses against different cancer cell types. 

## 5. Antiangiogenic Effects of Fucoidan *in Vivo*

Targeting tumor angiogenesis or new vasculature formation has advanced cancer therapy [44]. Limiting new blood vessel formation by antiangiogenic agents reduces intratumoral blood flow, hence limiting growth and metastatic potential. There have been contradictory reports of the effects of fucoidan on angiogenesis. Oversulfated fucoidan was found to inhibit tumor-induced angiogenesis [33,45], whereas fucoidan has been associated with neovascularization in other diseases [46].

Oversulfated fucoidan was found to inhibit the basic fibroblast growth factor (bFGF)-induced tube formation by human umbilical vein endothelial cells [45], while other sulfated polysaccharides inhibited the proliferation and migration of vascular endothelial cells by altering FGF binding to cell surface FGF receptors [47]. In contrast, fucoidan promoted FGF-2 effects *in vivo* [37] and LMW fucoidan (MW. *ca*. 4 kDa) prepared by radical degradation promoted bFGF-induced tube formation of endothelial cells [48,49]. On the other hand, in *ex vivo* angiogenesis assays, where rat aortic tissue was placed on Matrigel™ and capillary tube formation was measured, fucoidan derived from *Undaria pinnatifida* suppressed angiogenesis in the aortic rings when used at 100 µg/mL [50]. However, LMW fucoidan reduced intimal hyperplasia in a rat aortic allograft model of transplant atherosclerosis, while stimulating formation of an endothelial lining in the vascular allograft [51]. Wang and Miao reviewed the currently used marine-derived angiogenesis inhibitors, suggesting that different fucoidan preparations affect angiogenesis differently, depending on molecular weight and extent of sulfation: (1) LMW fucoidans (4–9 kDa) stimulated angiogenesis in different assays; (2) mid-molecular-weight fucoidans (15–20 kDa) enhanced HUVEC migration, but have not been shown to inhibit HUVEC tube formation; and (3) natural fucoidans of HMW (30 kDa) showed antiangiogenic properties by inhibiting proliferation, migration, and tube formation of endothelial cells and inhibiting vascular network formation [52].

Koyanagi *et al.* demonstrated that fucoidan prevented phosphorylation of the receptor for vascular endothelial growth factor (VEGF) upon VEGF binding [33]. Furthermore, the authors observed that repetitive intravenous administration of fucoidan at 5 mg/kg in mice suppressed neovascularization from surrounding blood vessels in the region adjacent to implanted Sarcoma 180 cells. Similarly, intraperitoneal administration of fucoidan (1 mg/mouse) in mice implanted with the murine plasma cell tumor line, MOPC-315, which expresses VEGF, reduced VEGF-induced angiogenesis, tumor neovascularization, and tumor growth [34]. In 2012, Xue *et al.* demonstrated that fucoidan caused a significant reduction in intratumoral VEGF expression in mice implanted with 4T1 breast cancer cells compared to untreated control animals [24]. These promising results indicate that fucoidan could play an important role as an antiangiogenic factor in cancer. Further investigations of angiogenesis using various *in vivo* cancer models treated with fucoidan are warranted. 

## 6. Mobilization of Hematopoietic Progenitor Cells

Reportedly, fucoidan can inhibit selectin function *in vitro* and *in vivo* [53]. Fucoidan blocks leukocyte rolling and interferes with various inflammatory responses in animal models [54,55,56,57,58]. In addition, it can reduce platelet aggregation by inhibiting P-selectin [59]. However, significant mobilization of progenitor cells and leukocytosis could be elicited in selectin-deficient mice similar to that of wild-type controls, suggesting that the mode of action of fucoidan is not through selectins [60,61]. The *in vivo* effects of fucoidan on leukocytes in other disease models are summarized in Table 2. 

**Table 2:** *In vivo* effects of fucoidan on leukocytes in various disease models.

Test	Route	Dose	Possible *in vivo* effects	References
Human	p.o.	330 mg	Mobilization of leukocytes	[12]
Human	p.o.	100 mg	Immune modulation	[15]
Rabbit	i.v.	10 mg/kg	Decreased influx of leukocytes into cerebrospinal fluid in meningitis	[58]
Mice	i.v. i.p.	50 mg/kg 50 mg/kg	Mobilization of hematopoietic progenitor stem cells (HPCs)	[61,62,63]
Rat	i.p.	25 mg/kg	Inhibition of extravasation of macrophages and CD4^+^ T cells to myocardium	[64]
Mice	p.o.	200 mg/kg	Th1 switch in *Leishmania* infection	[65]
Mice	i.p.	50 mg/kg	Improvement of pulmonary inflammation	[66]
Rat	i.p.	50 mg/kg	Inhibition of leukocyte infiltration in ischemic lesion	[67]
Mice	i.v.	10 mg/kg	Inhibition of infiltration of γδ T cells in pleural cavity	[68]
Mice	p.o.	0.05% (w/w) in mouse chow	Improvement of chronic colitis	[69]
Mice	i.v.	25 mg/kg	Inhibition of leukocyte rolling	[70]
Rat	p.o.	100 mg/kg	Decreased infiltration of neutrophils in myocardial infarct size	[71]
Cat	i.v.	25 mg/kg	Inhibition of leukocyte rolling	[72]

Interaction between stromal-derived factor-1 (SDF-1, CXCL12) and SDF-1α-binding chemokine (C-X-C motif) receptor 4 (CXCR4) is involved in the mobilization of hematopoietic progenitor stem cells (HPC), which are used after high-dose chemotherapy to support bone marrow regeneration [73]. In addition, CXCR4 is expressed on various cancer cell types [41,74], and the CXCL12–CXCR4 axis is involved in tumor progression, angiogenesis, metastasis, and survival [74,75,76]. Therefore, modulation of the CXCL12–CXCR4 axis by fucoidan seems an interesting target for cancer therapy. Fucoidan binds CXCL12, which is normally retained by heparan sulfate proteoglycans on the membrane of stromal cells or the extracellular matrix in bone marrow, thereby releasing CXCL12 into the circulation [60,77,78,79]. Negatively charged fucoidan seems to interact with basic residues of CXCL12 [80]. When mice were injected intravenously or intraperitoneally with 50 or 100 mg/kg of fucoidan, HPCs were mobilized from bone marrow [60,61,62], and plasma concentration of CXCL12 increased rapidly and dramatically [37,61,63]. In clinical trials, Irhimeh *et al.* demonstrated that oral fucoidan increased surface expression of CXCR4 on human CD34^+^ cells and the release of CD34^+^ cells from bone marrow to peripheral blood [12]. Therefore, future studies are warranted to determine whether fucoidan can affect the CXCL12–CXCR4 axis in tumor growth *in vivo*.

## 7. Role of Scavenger Receptor Type A in the Action of Fucoidan

Fucoidan is known to bind to various types of scavenger receptors (SR), including class A-, B-, and F-SRs [81,82,83]. Fucoidan has been used as an effective competitor for oxidized low-density lipoproteins in SR-A binding assays [84]. SR-A is mainly expressed in macrophages and DCs [85] and is implicated in changing the immune microenvironment in cancer. However, the reports on the role of SR-A in anticancer immunity have been contradictory. Wang *et al.* demonstrated that anticancer responses in SR-A^−/−^ mice were improved and correlated with an increased antigen-specific T cell response [86]. The authors showed that SR-A^−/−^ mice were highly responsive to inflammatory stimuli, such as lipopolysaccharide. Thus, the immunosuppressive role of SR-A in cancer might be due to its inhibition of proinflammatory responses by ligation of the toll-like receptor 4 (TLR4) rather than a direct inhibition of tumor immunity [87]. Indeed, it was shown that fucoidan, as a common SR-A ligand, also activated TLR4 signaling, and combined signaling through two distinct receptors resulting in a functional outcome not achieved by either receptor alone [88].

SR-A expression was lower in cancerous than in normal tissues and SR-A depletion was found to boost growth and angiogenesis of implanted Lewis lung carcinoma in mice [89]. Tumor cells caused SR-A up-regulation on macrophages [90], and elimination of SR-A-positive tumor-infiltrating leukocytes from the peritoneum of tumor-bearing mice relieved the T cell suppression and inhibited tumor growth [91,92]. Moreover, SR-A^−/−^ mice showed delayed growth of injected EL4 tumors and expression of inducible NO synthase, while showing significantly increased IFN-γ mRNA expression, suggesting that tumor-associated macrophages are highly active in SR-A-depleted conditions [93]. Recently, *in vitro* experimental results revealed that fucoidan can inhibit macrophage-induced tumor cell invasion [94]. Results obtained using an experimental autoimmune myocarditis model also showed that fucoidan administration attenuated progression of myocarditis by decreasing myocardial macrophage infiltration [64]. Therefore, we can speculate that macrophages are associated with immune suppression in tumor tissues and macrophage depletion in cancerous tissues by fucoidan may inhibit tumor progression. 

*In vitro* testing indicated that LMW fucoidan is internalized via endocytosis [38]. Recently, Zhu *et al.* demonstrated that fucoidan-SR-A internalization occurred through clathrin- and caveolae-dependent pathways [95]. They also revealed the mechanism involving the production of tumor necrosis factor (TNF)-α following SR-A binding by fucoidan. Fucoidan treatment of macrophages could promote recruitment of the major vault protein to lipid rafts to form an SR-A-major vault protein complex, leading to TNF-α production [96]. NO production by macrophages was significantly decreased in SR-A^−/−^ compared with the wild-type mice when macrophages were treated with fucoidan [97]. In addition, fucoidan abrogated SR-A-mediated chaperone uptake into macrophages [98] and DCs [83]. Our previous results also demonstrated that fucoidan decreased the binding of the anti-SR-A antibody to human blood DCs and failed to activate SR-A-depleted DCs [8]. These results suggest that the binding of SR-A by fucoidan leads to DC activation. Herber *et al.* demonstrated that DCs with high lipid content had low antigen-processing capacity and the frequencies of these cells were significantly increased in tumor-bearing mice and cancer patients [99]. Accumulation of lipids by DCs *in vitro* and *in vivo* was induced by tumor-derived factors that up-regulated SR-A expression on DCs. It was shown that SR-A overexpressing DCs could internalize modified lipoproteins from serum and fucoidan inhibited DC lipid uptake. Therefore, fucoidan might be effective in decreasing the frequency of lipid-laden and poorly antigen-presenting DCs in cancer patients by blocking SR-A, thus leading to an enhanced immune response. Consequently, further studies to reveal the possible effects of fucoidan on tumor growth in SR-A^−/^^−^ mice may help to unravel the immunological roles of fucoidan in cancer.

## 8. *In Vivo* Cytokine Production by Fucoidan in Other Diseases

In contrast to effects on cytokine expression in tumor-bearing mice, serum analysis of cytokines after fucoidan treatment has shown discrepant effects in several other disease models (Table 3). For example, splenic cytokine analysis in *Leishmania*-infected mice showed that fucoidan treatment at 200 mg/kg (p.o.) significantly increased levels of IFN-γ, IL-12, and TNF-α [65]. In an aspirin-induced stomach ulceration model, IFN-γ was increased, and levels of IFN-γ were further increased in rats treated with both aspirin and fucoidan at 400 mg/kg [100]. On the other hand, Maruyama *et al.* demonstrated that Mekabu-derived fucoidan suppressed production of Th2 cytokines in the bronchoalveolar lavage fluid after ovalbumin aerosol challenge [66]. Under normal conditions, intravenous treatment of mice and nonhuman primates with fucoidan was shown to increase levels of IL-8, monocyte chemoattractant protein 1, and matrix metalloproteinase 9 [60,63].

Other studies have shown that fucoidan suppresses cytokine levels in several disease models. Kang *et al.* demonstrated that treatment of rats with fucoidan at 50 mg/kg (i.p.) significantly decreased the number of the TNF-α-immunoreactive cells in the cerebral cortex and striatum, induced by lipopolysaccharide [67]. Concanavalin A-induced liver injury and a concomitant increase of plasma TNF-α and IFN-γ levels were prevented, but plasma IL-10 levels were increased by fucoidan treatment at 30 mg/kg (i.v.) [101]. Interestingly, the above mentioned inhibitory effects of fucoidan were reversed by pretreatment with an anti-mouse IL-10 antibody. Intravenous administration of fucoidan inhibited ovalbumin-induced γδ T cell accumulation in pleural cavities and lymph nodes in a murine model of ovalbumin-induced allergic pleurisy [68]. Moreover, pleural γδ T lymphocytes from fucoidan-treated mice showed reduced ovalbumin-induced IL-5 production, leading to decreased ovalbumin-induced eosinophil influx [68]. These results indicate that cytokine production profiles of fucoidan-stimulated immune cells in cancer patients may differ from those in patients with inflammatory or immune diseases.

## 9. *In Vivo* Antioxidant and Prooxidant Effects of Fucoidan

Prooxidant cytotoxic effects are important in clearing transformed cells from the body and limiting tumor growth. In contrast, oxidative stress is also associated with membrane lipid peroxidation, DNA damage, and mutagenesis, leading to tumor formation [102]. Fucoidan may have both antioxidant and prooxidant effects in cancer cells. We previously showed that fucoidan-induced apoptosis in leukemic cells was inhibited by glutathione and/or NAME addition [17]. Furthermore, fucoidan treatment of leukemic cells decreased intracellular glutathione concentrations and stimulated NO production. Zhang *et al.* demonstrated that fucoidan enhanced the apoptosis of cancer cells that responded to cisplatin, tamoxifen, or paclitaxel treatment via reduced glutathione levels, and enhanced production of intracellular reactive oxygen species in breast cancer cells [103]. According to Yang *et al.*, fucoidan isolated from *Undaria pinnatifida* induces the death of hepatocellular carcinoma cells by causing intracellular accumulation of high levels of reactive oxygen species, accompanied by damage to the mitochondrial ultrastructure, depolarization of the mitochondrial membrane potential, and caspase activation [23]. These results suggest that induction of oxidative stress may be an important event in fucoidan-induced cancer cell death.

**Table 3:** Changes of cytokine and growth factor levels by *in vivo* administration of fucoidan in cancer and other disease models.

Cytokines	*In Vivo* Changes	References
CXCL12	Increase in plasma	[37,51,61,63,80]
	Increase in myocardial ischemic tissue	[46]
IFN-γ	Increase in splenic T cells/A20 lymphoma	[32]
	Increase in splenic T cells/P-388	[40]
	Increase of secretion in plasma by aspirin	[100]
	Inhibition of increase in gastric ulcer lesion	[104]
TNF-α	Inhibition of increase in acute bacterial meningitis	[58]
	Inhibition of expression in ischemic lesion	[67]
	Inhibition of increase in ischemia-reperfusion injury	[71]
	Inhibition of increase in gastric ulcer lesion	[104]
	Inhibition of lipopolysaccharide-induced increase in brain	[105]
IL-1	Inhibition of increase in acute bacterial meningitis	[58]
IL-4	Decrease in bronchoalveolar lavage fluid	[66]
	Decrease in ovalbumin-sensitized spleen cells	[106]
IL-5	Inhibition of increase in pleural cavity of allergic pleurisy	[68]
IL-6	Decrease in plasma	[15]
	Inhibition of increase in colonic lamina propria of colitis	[69]
	Inhibition of increase in ischemia-reperfusion injury	[71]
IL-8	Increase in plasma	[63]
	Inhibition of lipopolysaccharide-induced increase in brain	[67,105]
IL-10	Increase in plasma level in liver injury	[101]
	Inhibition of decrease in ischemia-reperfusion injury	[71]
IL-12	Increase in splenic T cells/A20 lymphoma	[32]
	Increase in *Leishmania* infection	[65]
	Inhibition of increase in gastric ulcer lesion	[104]
MCP-1	Increase in plasma	[63]
VEGF	Reduction of mRNA expression in tumor tissues	[24]
	Increase in myocardial ischemic tissue	[46]
FGF-2	Potentiation of activity	[37]

In general, fucoidan preparations show antioxidant effects in other disease models *in vivo* [107,108,109] (summarized in Table 4). Pre-treating rats with fucoidan preparations caused suppression of lactate dehydrogenase and malondialdehyde levels, but caused normalization of superoxide dismutase, catalase, and glutathione peroxidase levels, decreasing necrosis and cirrhosis incidences in the liver of CCL_4_-treated rats [107]. Similarly, fibrosis and acetaminophen-induced liver injury were significantly suppressed by oral fucoidan intake [110,111]. Luo *et al.* investigated the effects of fucoidan on 1-methyl-4-phenyl-1,2,3,6-tetrahydropyridine (MPTP)-induced animal models of parkinsonism in C57/BL mice. The authors demonstrated that the administration of fucoidan at 25 mg/kg (i.p.) inhibited MPTP-induced lipid peroxidation and reduced the activities of antioxidant enzymes [108]. This fucoidan-induced alteration in antioxidant activity might lead to increased levels of striatal dopamine and its metabolite, increased expression of tyrosine hydroxylase, and reduced behavioral deficits. Suresh *et al.* demonstrated that fucoidan prevented increase of drug-metabolizing hepatic enzyme levels, which result from oxidative stress by diethylnitrosamine, a carcinogen in rats [30]. In contrast, splenocytes of fucoidan-treated mice infected with *Leishmania donovani* generated significantly high levels of superoxide and NO [65].

**Table 4:** *In vivo* effects of fucoidan on other diseases related to the oxidant production.

Test	Route	Dose	Possible *in vivo* effects	References
Mice	i.p.	25 mg/kg 15 mg/kg	Prevention of MPTP-induced neurotoxicity Prevention of lipopolysaccharide-induced neurotoxicity	[108] [105]
Mice	i.p.	25 mg/kg	Neuroprotection via antioxidant activity	[108]
Mice	i.p.	100 mg/kg	Prevention of ischemia-reperfusion injury	[112]
Rat	p.o. i.p.	100 mg/kg	Suppression of liver fibrogenesis and drug-induced liver injury	[110,111]

Similar to fucoidan, green tea has shown both antioxidant and prooxidant effects in cancer cells [113]. Prooxidant effects of green tea apparently cause tumor cell apoptosis and also induce endogenous antioxidant mechanisms that protect against carcinogenic insults in normal tissues [113]. Fucoidan has antioxidative and prooxidative potential in animal models of renal ischemia-reperfusion injury, however, fucoidan alone did not affect malondialdehyde and superoxide dismutase levels in the sham-operated group [112]. Recently, it was shown that intracellular and secreted H_2_O_2_ from HT1080 human fibrosarcoma cells were both greatly repressed upon tumor cell treatment with enzyme-digested fucoidan preparations extracted from seaweed Mozuku of *Cladosiphon novae-caledoniae kylin* [114]. However, Ye *et al.* suggested that H_2_O_2_ release by cancer cells is one of the triggering factors to promote angiogenesis rather than apoptosis [114]. Therefore, more careful *in vivo* studies using highly purified fucoidan preparations are essential to determine whether fucoidan has an antioxidant or prooxidant role in cancer prevention and treatment.

## 10. Conclusions

Fucoidan shows promising characteristics that warrant further development of the substance as a future marine drug. Recent *in vivo* studies suggest fucoidan to be a potential preventive or therapeutic agent for controlling cancers. However, the *in vitro* and *in vivo* mechanisms underlying the observed anticancer effects of fucoidan have not been fully investigated. The anticancer effects of fucoidan *in vivo* may be due to the inhibition of tumorigenesis and metastasis. More importantly, fucoidan may act by promoting immune responses or antiangiogenesis in tumor tissues. The mobilization of hematopoietic progenitor cells by fucoidan is yet another interesting characteristic of the substance that harbors therapeutic potential. These findings need to be corroborated by further preclinical studies. Orally delivered fucoidan appears promising as a marine drug in several diseases [2], but fucoidan administration via other routes is also possible. However, fucoidan preparations isolated from different sources have shown differential anticancer effects *in vivo* because of correspondingly different structural properties. Therefore, as a next step, the determination of the structural characteristics responsible for the *in vivo* anticancer activities of fucoidan will be essential for its potential use as a marine drug.
